# Strand-specific effect of Rad26 and TFIIS in rescuing transcriptional arrest by CAG trinucleotide repeat slip-outs

**DOI:** 10.1093/nar/gkab573

**Published:** 2021-07-01

**Authors:** Jun Xu, Jenny Chong, Dong Wang

**Affiliations:** Division of Pharmaceutical Sciences, Skaggs School of Pharmacy & Pharmaceutical Sciences; University of California, San Diego, La Jolla, CA 92093, USA; Division of Pharmaceutical Sciences, Skaggs School of Pharmacy & Pharmaceutical Sciences; University of California, San Diego, La Jolla, CA 92093, USA; Division of Pharmaceutical Sciences, Skaggs School of Pharmacy & Pharmaceutical Sciences; University of California, San Diego, La Jolla, CA 92093, USA; Department of Cellular and Molecular Medicine, University of California, San Diego, La Jolla, CA 92093, USA; Department of Chemistry and Biochemistry, University of California, San Diego, La Jolla, CA 92093, USA

## Abstract

Transcription induced CAG repeat instability is associated with fatal neurological disorders. Genetic approaches found transcription-coupled nucleotide excision repair (TC-NER) factor CSB protein and TFIIS play critical roles in modulating the repeat stability. Here, we took advantage of an *in vitro* reconstituted yeast transcription system to investigate the underlying mechanism of RNA polymerase II (Pol II) transcriptional pausing/stalling by CAG slip-out structures and the functions of TFIIS and Rad26, the yeast ortholog of CSB, in modulating transcriptional arrest. We identified length-dependent and strand-specific mechanisms that account for CAG slip-out induced transcriptional arrest. We found substantial R-loop formation for the distal transcriptional pausing induced by template strand (TS) slip-out, but not non-template strand (NTS) slip-out. In contrast, Pol II backtracking was observed at the proximal transcriptional pausing sites induced by both NTS and TS slip-out blockage. Strikingly, we revealed that Rad26 and TFIIS can stimulate bypass of NTS CAG slip-out, but not TS slip-out induced distal pausing. Our biochemical results provide new insights into understanding the mechanism of CAG slip-out induced transcriptional pausing and functions of transcription factors in modulating transcription-coupled CAG repeat instability, which may pave the way for developing potential strategies for the treatment of repeat sequence associated human diseases.

## INTRODUCTION

Genomic instability of ubiquitous simple sequence repeats, such as trinucleotide repeat expansion and contraction, is associated with >50 genetic disorders in humans (1,2). In particular, CAG trinucleotide repeat expansion beyond a pathological threshold is linked to over ten neurodegenerative disorders, such as Huntington's disease, myotonic dystrophy, and several kinds of spinocerebellar ataxias (3). In general, longer repeat expansions lead to earlier onset and greater severity of the diseases, suggesting a common element to the repeat instability mechanism (4,5).

Trinucleotide DNA repeats have been shown to form slipped-strand, non-B-form secondary structures (slip-out), in which the complementary DNA strands display an out-of-register alignment (6). CAG repeats have been shown to form stable slip-out structures containing mismatched hairpins both biochemically (7,8) and structurally (6), and these slip-out structures are believed to be the critical mutagenic intermediates during CAG repeat instability (2,9,10). In support of this notion, the propensity of the repeat DNA to form secondary structures is strongly correlated with the probability of repeat instability during DNA replication (11,12), repair (13–16), and transcription (17,18).

Several lines of evidence support that transcription plays a critical role in CAG repeat instability. First, CAG repeat length heterogeneity in non-dividing neural tissues increases with the age of the patient, indicating that somatic repeat instability could occur in the absence of DNA replication (19,20). Second, increased transcription level promotes CAG repeat instability in *Escherichia coli* (21), yeast (22) and human cells (17,23). Third, naphthyridine-azaquinolone (NA), a small compound that specifically binds slipped-CAG DNA, induced repeat instability is independent of proliferation/ replication, yet dependent on transcription through the repeat (24). Lastly, deletions of transcription elongation and repair factors, such as CSB, significantly alter the triplet repeat stability (17,25,26).

Transcription of the CAG-repeat sequences promotes the formation of slip-out DNA structures, and these slip-out structures have been shown to induce Pol II pausing through an under-characterized mechanism (27,28). The number of CAG repeat is strongly correlated with disease progression, in which a higher CAG repeat number is indicative of a more severe and earlier onset of disease symptoms (4). Thus, studying how different lengths of CAG repeat slip-outs affect transcription elongation is of great interest to understand the relationship between repeat number and disease progression.

Transcription elongation is a tightly regulated process and transcriptional arrest triggers the transcription-coupled nucleotide excision repair (TC-NER) pathway to removes transcription-blocking DNA lesions (29–31). Intriguingly, a variety of transcription elongation factors have dual roles in facilitating Pol II transcription elongation through the genome (32) and regulating TC-NER. CSB and its yeast ortholog Rad26 are SWI2/SNF2 family ATPases that play critical roles in transcription elongation and TC-NER (33), and they are the first proteins to be recruited to DNA damage-stalled Pol II during TC-NER(34). Mutations in the CSB gene have been shown to cause neurological diseases (35). TFIIS is a transcription elongation factor that stimulates backtracked transcript cleavage by the polymerase active site and resumes transcript elongation (36). It promotes Pol II bypass of nucleosome barriers (37,38) and several small DNA damage-induced Pol II pausing (39).

Although it has been proposed that the CAG slip-out induced Pol II arrest may trigger TC-NER (27,40,41) and CSB/Rad26 and TFIIS play an important role in transcription-coupled CAG repeat instability in genetic studies (17,25,42), several key questions remain elusive: What is the mechanism of Pol II arrest by CAG slip-out? What is the function of CSB/Rad26 and TFIIS in modulating how Pol II transcribes CAG slip-out templates? What is the implication of the *in vitro* study results for the *in vivo* repeat instability?

Here, we used an *in vitro* reconstituted system with purified proteins from budding yeast to address these questions. First, we showed that CAG slip-out has a length-dependent and strand-specific effect on Pol II transcription. The TS slip-out structures lead to both proximal and distal transcriptional pausing, whereas the NTS slip-out structures only cause proximal transcriptional pausing. We found that R-loop formation plays a specific role in distal pausing sites of TS slip-out, but not in the proximal pausing sites of the TS or NTS slip-out. In contrast, translocation blockage induced backtracking that correlates with both NTS and TS slip-out induced proximal pausing. Strikingly, we uncovered strand-specific roles of Rad26 and TFIIS in modulating Pol II bypass of CAG slip-out transcriptional barriers. Taken together, our biochemical data provide new mechanistic insights to understand how slip-out structures induce transcriptional arrest and the functions of Rad26/CSB and TFIIS in modulating CAG repeat induced transcriptional arrest and transcription-coupled CAG repeat instability.

## MATERIALS AND METHODS

### Protein expression and purification

*Saccharomyces cerevisiae* 10-subunit Pol II was purified essentially as previously described (43). Briefly, Pol II with protein A tag in the Rpb3 subunit was purified by an IgG affinity column (GE Healthcare), followed by Hi-Trap Heparin (GE Healthcare) and Mono Q anion exchange chromatography columns (GE Healthcare). Recombinant Rpb4/7 heterodimer was purified from *Escherichia coli* by Ni-affinity chromatography, followed by gel filtration as previously described (44). Overexpression and purification of Rad26 were performed essentially as previously described (34). Expression and purification of yeast TFIIS were prepared as described (45). Recombinant Spt4/5 was expressed and purified essentially as described (46). Expression and purification of yeast Elf1 were performed as previously described (47).

### Generation of non-B form slip-out DNA templates

Preparation of the DNA templates were performed essentially as previously described (48). Briefly, TS (template strand) and NTS (non-template strand) were annealed in 10 mM Tris–HCl (pH 7.5) and 5 mM MgCl_2_ with an equal molecular ratio of each complementary single-stranded oligonucleotide for 10 min at 95°C, followed by slow cooling (15 h) to 23°C and subsequent native PAGE purification. Annealing of the oligonucleotide strands resulted in 9-nt sticky ends for ligation to the downstream of the elongation complex. The DNA/RNA sequences used in this study are shown in Supplementary Table S1.

### Generation of slip-out DNA containing elongation complex

Pol II elongation complex (EC10) was assembled essentially as previously described (34). First, radiolabeled 10-mer RNA was annealed to the template strand DNA, followed by incubation with Pol II for 10 min at room temperature (23°C) and then 2 min at 37°C. To this, biotin-labeled non-template strand DNA was added and incubated for 5 min at 37°C, followed by 20 min at room temperature (23°C). The assembled elongation complex was incubated with streptavidin magnetic beads (NEB) for 30 min at room temperature (23°C) and subsequently washed with elongation buffer (EB) (20 mM Tris–HCl (pH 7.5), 5 mM MgCl_2_, 40 mM KCl, 5 mM DTT). The immobilized elongation complex was ligated to the downstream slip-out DNA and washed two times with EB buffer. Next, Rpb4/7 was added to a final concentration of 5 μM and incubated for 10 min at 23°C to generate 12-subunit elongation complex, followed by washing three times with EB buffer to remove excess Rpb4/7.

### *In vitro* transcription assay

Transcription reaction was started by adding 1 mM rNTPs (or specifically indicated in the figure legends) with an additional 3 mM dATP to support Rad26 ATPase activity. The concentration for Rad26 and TFIIS are 100 nM. Reactions were performed at 30°C and allowed to continue for the desired time point and then quenched by adding an equal volume of stop buffer (90% formamide, 50 mM EDTA, 0.05% xylene cyanol and 0.05% bromophenol blue). Samples were denatured for 15 min at 95°C in formamide loading buffer, and the extended RNA was separated from the unextended RNA by denaturing urea PAGE (6 M urea). The gels were visualized by phosphorimaging and quantified using Imagelab software (Bio-Rad). All bands above the ligation truncations (except the transcript from the ligation truncation, labeled with asterisk) were used in quantification. Bands beneath the run-off bands but above the ligation truncation bands are grouped as ‘blockage bands’ for quantification. The percentage of bypass was calculated as intensity of run off bands/total intensity above the ligation truncation.

## RESULTS

### CAG slip-outs impair Pol II transcription elongation in a length-dependent and strand-specific manner

**Figure 1.**
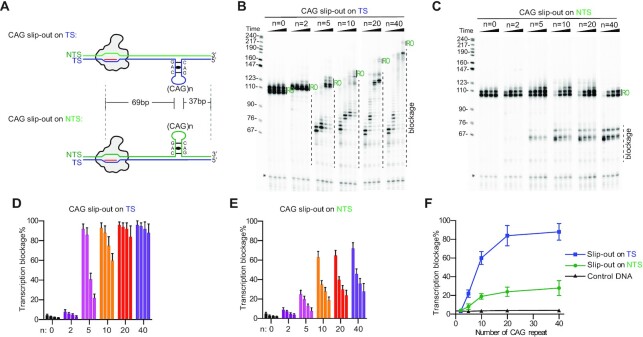
CAG slip-out induces length-dependent and strand-specific transcriptional pausing by Pol II. (**A**) Scheme of Pol II EC with a TS and an NTS CAG slip-out. (**B**) CAG slip-out on the TS strand blocks Pol II transcription elongation. (**C**) CAG slip-out on the NTS strand leads to Pol II transcriptional pausing. Markers shown on the left are a MspI digest of pBR322 DNA plasmid. The expected run-off bands are labeled as RO in green. Ligation truncation is indicated by asterisks. (**D, E**) Quantitative analysis of the time courses of CAG repeat induced Pol II arrest. The numbers of CAG repeat are shown at the bottom. Time points are 0.3, 1, 3 and 10 min, respectively. (**F**) The CAG slip-out induced transcription blockage is strand-specific and length dependent. The last time point (10 min) results from D and E are plotted. All data in this figure are obtained and quantified from three independent experiments (means ± SEM, *n* = 3).

We previously showed that B-form duplex and non-B form slip-out of CTG•CAG repeat can cause Pol II pausing in a very distinct manner (48). Strikingly, we found that Spt4/5 is able to promote transcription bypass of B-form CAG duplexes, but inhibits transcription from non-B slip-outs (48). To further understand the underlying mechanism of CAG slip-out induced transcriptional pausing/arrest, we first investigated the effects of different lengths of preformed CAG slip-out on the template strand (TS) or non-template strand (NTS) on Pol II transcription elongation using our defined system (Figure 1A). We found that CAG repeat slip-outs impair Pol II transcription elongation in a length-dependent and strand-specific manner. While Pol II was able to efficiently bypass the controlled scaffolds with no slip-out insertions (*N* = 0) and short slip-out (CAG)_2_ repeats and obtain run-off transcript (Figure 1B and C), we observed multiple pausing sites when Pol II encounters five or more CAG slip-outs on either TS or NTS. By contrast, Pol II was able to bypass the same length of random DNA (Supplementary Figure S1). The amount and strength of transcriptional pausing/stalling are well correlated with the length of CAG repeats (Figure 1B and C). For example, we observed almost 100% transcriptional pausing induced by TS (CAG)_5_ template at 20 s, and most of these transcriptional pausing transcripts can be further extended with longer incubation time (10 min). In sharp contrast, we observed persistent strong pausing on template strand containing TS (CAG)_20_ and (CAG)_40_ slip-outs. This result recapitulates the effect of CAG repeat expansion seen in its associated patients (10,49). Run-off RNA products often contained several closely spaced bands, probably due to either increased probability of spontaneous transcription termination close to the end of substrates or template-independent addition of nucleotides to the run-off transcript. Nevertheless, it does not affect the interpretation of our results, and this heterogeneity is not caused by CAG slip-outs, as it is also shown in the control template (*N* = 0).

The strength of CAG slip-outs induced transcriptional pausing/stalling is strand-specific (Figure 1D–F). Overall, the CAG slip-outs lead to much stronger transcriptional pausing when they are located at the TS strand than at the NTS strand. For example, with the same (CAG)_40_ slip-outs, we observed over 90% of Pol II remain in persistent stalling induced by the TS (CAG)_40_ slip-out structure (at 10-minute time point), whereas we found most of Pol II slowly bypass NTS (CAG)_40_ slip-out induced pausing and only 28% of Pol II remain stalled after 10-minute incubation. The pattern of CAG slip-out induced transcriptional pausing/stalling is also strand-specific (Figure 1B and C), which is also distinct from the transcriptional pausing induced by CTG•CAG duplex (48). We observed that for TS-CAG slip-out induced transcriptional pausing, the RNA transcripts at proximal pausing (lower bands) can be slowly extended to the longer RNA transcripts at distal stalling sites (upper bands) with a feature of ladder-like consecutive bands over time (Supplementary Figure S2). In contrast, the NTS-CAG slip-out induced transcriptional pausing bands are two or three defined bands that are separated by ∼5–10 nts and are relatively stable (Figure 1B and C).

### Pol II backtracking and R-loop formation correlate with different types of CAG induced transcriptional arrest

**Figure 2.**
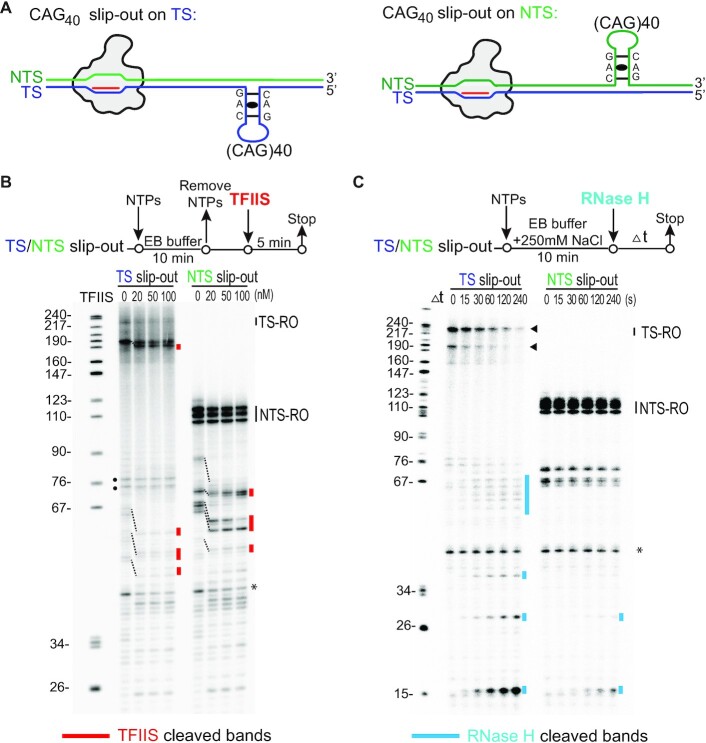
Analysis of Pol II EC paused on TS or NTS CAG slip-out templates. (**A**) Scheme of Pol II EC used in this figure. (**B**) CAG slip-outs on TS and NTS induce Pol II backtracking. Scheme of the experimental setup is shown at the top. TFIIS was used as a probe to detect the state of Pol II EC and was present at lower concentrations that do not induce Pol II backtracking, as evidenced by the lack of its effect on the length of multiple arrested transcripts (indicated by black dots). The red lines indicate TFIIS cleaved transcript. Dashed line shows the changes before and after TFIIS cleavage. (**C**) Transcription from TS CAG slip-outs, but not NTS CAG slip-outs, specifically causes R-loop formation. A final concentration of 250 mM of NaCl was added to the system to increase the specificity of RNase H. High molecular weight transcript from CAG repeat on TS (indicated by a black triangle) was cleaved by RNase H. The blue lines indicate RNase H digested transcript. The asterisks indicate the RNA transcript from template with incomplete ligation (ligation truncation).

Most long-lived transcriptional pausing and arrests are caused by RNA polymerase backtracking (50). TFIIS stimulates the cleavage activity of Pol II and shortens the backtracked RNA transcript (34,36), but it cannot cleave RNA transcripts when Pol II EC is in a post-translocation state. We therefore used TFIIS as a probe to detect the translocation states of Pol II EC and investigated whether the Pol II ECs arrested by CAG slip-outs are backtracked. To this end, (CAG)_40_ slip-out was chosen as the model system (Figure 2A), because it is above the clinical threshold that leads to repeat instability and pathogenesis and induces strong pausing in our *in vitro* reconstituted system (Figure 1B, C) (5,51). As shown in Figure 2B, (CAG)_40_ slip-out induced Pol II arrested complexes were generated by incubating rNTPs for 10 minutes, followed by the removal of unincorporated rNTPs (to avoid reincorporation of rNTPs after TFIIS cleavage), and low concentrations of TFIIS was added to the system. We found that TFIIS shortens the transcripts corresponding to proximal stalling bands induced by TS slip-outs and almost all stalling bands induced by NTS slip-outs (dashed lines, Figure 2B), indicating that Pol II backtracking is a universal feature for these arrested complexes when they encounter the CAG slip-outs regardless of TS or NTS strand location.

Intriguingly, we noticed two major distal stalling bands induced by TS slip-outs are resistant to TFIIS stimulated cleavage (two black dots, Figure 2B), suggesting a distinct type of stalled complexes that is not backtracked. This observation promoted us to consider an alternative mechanism that leads to Pol II pausing/stalling. Aside from Pol II backtracking, extended RNA/DNA hybrid (R-loop) formation can also lead to transcriptional pausing/arrest (41,52,53). Note that R-loop formation and Pol II backtracking are two mechanistically-distinct, but not mutually exclusive, processes during transcription. Importantly, previous studies suggested R-loop formation is involved in transcription-coupled CAG repeat instability in yeast (22) and human cells (54). An *in vitro* study with purified single-subunit viral RNA polymerases (T3, T7 and SP6) identified transcription of CTG•CAG repeats induces R-loop formation (54,55), and further processing of the R-loop induces its instability (18). These results promoted us to test whether R-loop was generated in our reconstituted Pol II transcription system. We post-treated RNA transcripts with RNase H, which specifically digests the RNA strand in an RNA/DNA heteroduplex (Figure 2C). Intriguingly, we found that transcripts from TS CAG slip-outs were very sensitive to RNase H digestion, whereas the majority of transcripts from NTS CAG slip-outs were resistant to RNase H digestion and only a minimum of background level of transcript can be digested by RNase H (Supplementary Figure S3). These results reveal that R-loop is preferentially formed from the transcription of TS CAG slip-outs versus NTS CAG slip-outs. Taken together, our experiments revealed distinct and strand-specific Pol II arrest by CAG slip-outs: strong and persistent distal Pol II arrest by TS slip-out is featured by R-loops formation, whereas moderate Pol II pausing by NTS slip-out or proximal pausing by TS slip-out are featured by Pol II backtracking.

### Rad26 promotes Pol II bypass of the NTS (CAG)_40_ but not TS (CAG)_40_ slip-out

**Figure 3.**
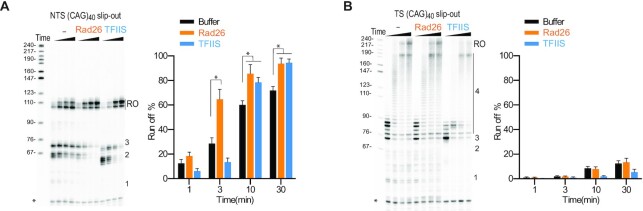
Strand-specific effects of Rad26 and TFIIS on transcriptional bypass of slip-out structures. (**A**) Effect of Rad26 and TFIIS on transcriptional bypass of NTS slip-out structures. (**B**) Effect of Rad26 and TFIIS on transcriptional bypass of TS slip-out structures. All data in this figure were obtained and quantified from three independent experiments (means ± SEM, *n* = 3, two-tailed Student’s t-test, *P < 0.05).

CSB promotes transcription elongation and is involved in transcription-induced CAG repeat instability in genetic studies (25). To further understand the functions of CSB in modulating transcriptional arrest by CAG repeat slip-outs, we assessed the effects of Rad26, the yeast ortholog of CSB, on promoting transcriptional bypass of CAG slip-out structures (Figure 3).

First, we evaluated the effect of Rad26 on Pol II transcriptional bypass of NTS (CAG)_40_ slip-out (Figure 3A). The addition of Rad26 greatly reduced transcriptional pausing at the translocation blockage sites (positions 1, 2, 3) and significantly increased the run-off product (Figure 3A). Strikingly, in contrast to the strong effect of promoting Pol II bypass of NTS (CAG)_40_ slip-out, the addition of Rad26 has little effect on promoting Pol II bypass of the TS (CAG)_40_ slip-out (Figure 3B).

### TFIIS promotes Pol II bypass of the NTS (CAG)_40_ but inhibits TS (CAG)_40_ slip-out bypass

TFIIS also has a dual role in transcription elongation and transcription-induced CAG repeat instability (25). To further evaluate the function and understand the underlying mechanism, we assessed the effects of TFIIS on transcription bypass of DNA slip-out structures (Figure 3). Interestingly, TFIIS reduces both NTS and TS slip-outs induced transcriptional pausing at the proximal pausing sites (positions 1, 2, 3) (Figure 3A, B). TFIIS significantly increases the run-off product on the transcriptional bypass of NTS (CAG)_40_ slip-out (Figure 3A). However, surprisingly, TFIIS inhibits bypass of TS (CAG)_40_ slip-out and no run-off product was observed in the presence of TFIIS (Figure 3B).

### Rad26 and TFIIS overcome the inhibitory effect of Spt4/5 and Elf1

**Figure 4.**
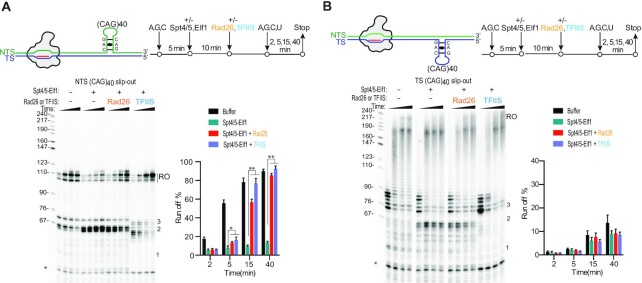
Rad26 and TFIIS overcome the inhibitory effect of Spt4/5 and Elf1 and promote overall transcriptional bypass of slip-outs in a strand-specific manner. (**A**) For NTS CAG-slip-out, Rad26 and TFIIS overcome the inhibitory effect of Spt4/5 and Elf1 and promote overall transcriptional bypass of NTS CAG slip-out. (**B**) For TS CAG slip-out, Rad26 and TFIIS overcome the inhibitory effect of Spt4/5 and Elf1 at proximal pausing sites, but failed to promote Pol II bypass of the R-loop induced transcription pausing. All data in this figure were obtained and quantified from three independent experiments (means ± SEM, *n* = 3, two-tailed Student's *t*-test, *P < 0.05, **P < 0.01, NS, not significant).

Our recent results show that the presence of transcription elongation factors Spt4/5 and Elf1 greatly inhibits Pol II bypass of the slip-out structures induced translocation blockage (48). To test whether Rad26 and TFIIS can overcome the inhibitory effect of Spt4/5 and Elf1, we assessed the roles of Rad26 and TFIIS in modulating the transcriptional arrest of Pol II-Spt4/5-Elf1 elongation complex induced by CAG slip-outs (Figure 4). We found that both Rad26 and TFIIS modulate the inhibitory effect of Spt4/5 and Elf1 on transcription processing of CAG slip-out template in a strand-specific manner. Interestingly, we also observed the inhibitory effect of Spt4/5 and Elf1 is strand-specific (Supplementary Figure S4). For the NTS CAG slip-out template, the Spt4/5-Elf1 complex has a much stronger inhibitory effect than that of TS slip-out. Intriguingly, both Rad26 and TFIIS can effectively counteract the strong inhibitory effect of Spt4/5-Elf1 and significantly increase the overall run-off product from the NTS CAG slip-out template (Figure 4A). In particular, TFIIS dramatically reduced NTS CAG slip-out induced Pol II proximal pausing at the translocation blockage sites (positions 1, 2, 3). By contrast, for the TS CAG slip-out template, Rad26 and TFIIS failed to increase the TS run-off product (Figure 4B), although Rad26 marginally and TFIIS prominently reduced proximal pausing translocation blockage at position-2 (Figure 4B and Supplementary Figure S5).

## DISCUSSION

### Strand-specific molecular mechanisms of CAG-induced transcriptional arrest

Transcription plays critical roles in CAG repeat instability, especially in the non-dividing neuron cells. While transcription itself does not change the DNA sequence, transcriptional arrest causes the collisions of transcription machineries and replication machineries, and DNA repair processes that lead to the repeat instability. Both CSB and TFIIS play pivotal roles in modulating transcriptional pausing/arrest induced by a variety of DNA lesions and nucleosome barriers (34,56–58). However, the underlying mechanism of CAG repeat induced Pol II transcriptional pausing/arrest and the functions of CSB and TFIIS in the regulation of Pol II transcriptional pausing remain elusive. Therefore, reconstituting a defined system for eukaryotic Pol II transcription and analyzing the roles of individual transcription/repair factors in Pol II pausing/processing of the CAG repeat is imperative.

Here, we systematically studied the effect of slip-out DNA structures on Pol II elongation using purified proteins and DNA/RNA scaffold. We found the inhibitory effect of CAG slip-out on transcription is length-specific and strand-specific. We observed stronger transcriptional arrest with CAG slip-outs containing longer CAG repeats. We found that Pol II is able to efficiently bypass two CAG repeats but pauses at multiple pausing sites when it encounters CAG slip-outs containing five or more CAG repeats on either TS or NTS. Importantly, we observed persistent strong pausing on TS (CAG)_20_ and (CAG)_40_ slip-out template which recapitulates the effect of CAG repeat expansion seen in its associated patients (10,49). With the same repeat number, the CAG repeat slip-outs on TS have a stronger blocking effect than the slip-outs on NTS. Further biochemical analysis revealed that while both NTS and TS slip-out induce Pol II backtracking at the proximal pausing sites, the TS slip-out preferentially promotes R-loop formation and induces Pol II stalling at distal pausing sites. Indeed, when Pol II slowly moves from the proximal pausing sites to the distal pausing sites, the imperfect hairpin of CAG slip-out region partially unwinds and serves as the template for RNA extension (Supplementary Figure S2). The nascent RNA can also readily form a stable extended RNA/DNA hybrid (R-loop) with newly exposed CAG repeat region. R-loop is preferentially formed at TS CAG slip-out as there is no complementary segment of non-template DNA to compete off the RNA:DNA in this region (Supplementary Figure S2). Our finding that R-loop is preferentially formed in the transcription of TS CAG slip-out at distal pausing sites therefore provide mechanistic insights into the understanding of R-loop formation observed from previous studies (22,54,55,59).

### Diverse roles of elongation factors in transcriptional processing of CAG repeats

Transcription factors Rad26, TFIIS, and Spt4/5 have been shown to promote Pol II transcription elongation on naked DNA (34,47,60) or nucleosomal templates (38,61). Elf1 is a transcription elongation factor (62) that works synergistically with Spt4/5 to promote transcription bypass of nucleosome (61) and plays an important role in TC-NER (31). Our studies provide a comprehensive understanding of the roles of these factors in modulating Pol II transcription of CAG repeat slip-out.

**Figure 5.**
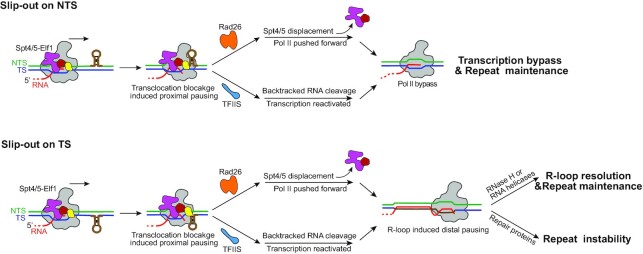
Model of the functions of CSB/Rad26, TFIIS, and Spt4/5-Elf1 in transcription-dependent CAG repeat instability. For NTS CAG slip-out, Rad26 and TFIIS overcome the inhibitory effect of Spt4/5 and Elf1 on transcription bypass of NTS CAG slip-out induced translocation blockage and result in repeat maintenance. For TS CAG slip-out, Rad26 and TFIIS overcome the inhibitory effect of Spt4/5 and Elf1 on transcription bypass of TS CAG slip-out induced translocation blockage but are not able to facilitate Pol II bypass of the R-loop induced transcription pausing. If R-loop can be resolved in a timely manner by RNase H or RNA helicases, repeat is maintained. Alternatively, persistent R-loop cause transcription-replication collision and genome instability. Recruitment of repair factors often results in repeat instability.

Importantly and surprisingly, the effects of the transcription factors on slip-out structures are factor-specific and strand-specific. In this study, we showed that the effect of Rad26 and TFIIS on slip-out induced Pol II transcriptional arrest are strand-specific. Both Rad26 and TFIIS can promote Pol II bypass of NTS slip-out structures, but not TS slip-out structures. We recently reported that elongation factor Spt4/5 along with Elf1 strongly inhibit Pol II bypass of both TS and NTS slip-outs structures, which is in sharp contrast to their canonical roles in promoting Pol II elongation processivity in naked B-form DNA duplex or nucleosome substrates (48). These findings also lead to an open question: How is the CAG slip-out induced transcriptional arrested Pol II-Spt4/5-Elf1 rescued? The Pol II binding sites for Spt4/5 and Rad26 are largely overlapping (Supplementary Figure S6), indicating the binding of these factors are mutually exclusive, and a possible role of Rad26 as an antagonist of the repression of Pol II bypass of the CAG slip-outs by Spt4/5 and Elf1 (Figure 5). Indeed, for the NTS CAG slip-out template, we found that Rad26 and TFIIS were able to overcome the inhibitory effect of Spt4/5 and Elf1 on Pol II arrest at the proximal translocation blockage sites (Figure 4). The rescue mechanisms of TFIIS and Rad26 are different (band patterns indicated in Figure 4A). Since Pol II is backtracked at the translocation blockage sites (Figures 3) and cleaved in the presence of TFIIS (lower bands observed near sites 1–3 in Figure 4A), we propose the mechanism of TFIIS overcoming the inhibitory effect of Spt4/5-Efl1 is through cleavage of the backtracked RNA and reactivating the arrested Pol II to have a second chance to bypass the barriers (Figure 5). In contrast, Rad26 binds behind Pol II and promotes Pol II forward translocation by its ATP-dependent DNA translocase activity. Sp4/5 is displaced by Rad26 and therefore the inhibitory effect of Spt4/5 is released (Figure 5) (34,56). For the TS CAG slip-out template, in striking contrast, we observed that Rad26 and TFIIS failed to rescue these transcriptional arrest (Figure 4B). These strand-specific effects of Rad26 and TFIIS in transcription processing of CAG slip-outs may have implications in CAG fragility and instability (see discussion below).

### Implications of roles of Rad26/CSB and TFIIS for CAG fragility and instability

Our biochemical results provide important insights into understanding the functions of the transcription factors in transcription-coupled CAG repeat instability in vivo (Figure 5). A previous study reported that knockdown of CSB and TFIIS reduces CAG repeat contraction in human cells (25). Consistently, CSB knockout in mice leads to a significant increase in CAG repeat expansion and reduction in contraction (26). Moreover, CSB has been shown to protect against CGG repeat expansion in another mice study (63). Interestingly, deletion of Rad26 leads to a 2.4-fold increase of (CAG)_70_ fragility (breakage) and a modest 2-fold increase of contraction of (CAG)_70_ in budding yeast (22). Altogether, these previous results indicate Rad26/CSB and TFIIS play a protective role in CAG repeat expansion by preventing repeat expansion and/or promoting repeat contraction. Here we uncovered that Rad26 and TFIIS facilitate Pol II bypass of CAG NTS slip-out structures. These two factors have distinct mechanisms in promoting Pol II bypass: Rad26/CSB facilitates Pol II forward translocation in an ATP-dependent manner, whereas TFIIS helps the cleavage of backtracked transcripts and reactivate Pol II complex into a new active state for transcription elongation. Because repair of the NTS slip-out often results in repeat expansion (25,27), we speculate that Rad26/CSB and TFIIS promote Pol II bypass of the NTS slip-out and reduce persistent transcriptional arrest and subsequent genome instability and DNA repeat expansion. Therefore, Rad26/CSB and TFIIS may prevent transcription-coupled genome instability when there is an NTS CAG slip-out (Figure 5).

In this study, we also revealed that Rad26 and TFIIS are unable to promote complete Pol II bypass of the long TS (CAG)_40_ slip-out (Figure 4). Although they can facilitate Pol II to bypass the proximal pausing induced by translocation blockage (Figures 4 and 5), they are not able to rescue arrested Pol II from the R-loop induced distal pausing in long TS CAG slip-out. This result also has important implications in understanding the roles of Rad26 and TFIIS in CAG repeat stability. By preventing Pol II pausing at proximal pausing sites, Rad26 and TFIIS facilitate Pol II to run further into the CAG repeat template. Therefore, for short CAG repeat/slip-out, Rad26 and TFIIS can facilitate run-off bypass. On the other hand, for long CAG repeat/slip-out that they cannot help Pol II fully go through, Rad26 and TFIIS may promote the accumulation of long, stable R-loops. If R-loop can be resolved in a timely manner, such as by RNase H or RNA helicases, CAG repeat is maintained (5,41,64,65). Alternatively, persistent R-loop can lead to transcription-replication collision and genome instability (5,41,64,65). Indeed, several TC-NER factors, including CSB (human homolog of Rad26) and XPG, are reported to be involved in R-loop processing (66–68). XPG excises R-loop non-canonically to generate DSB and cause genome instability (66–68). Interestingly, several previous studies suggest that the transcription-coupled R-loop formation causes CAG repeat instability (mostly contraction) that are involved with NER and BER repair factors (22,25,59). We predict that long, stable R-loop formation, promoted by Rad26 and TFIIS, may subsequently trigger the repair of TS CAG repeat and result in repeat contraction (Figure 5). The precise transcription-coupled repair mechanism of TS CAG-slip-out remains an open question and awaits further studies in the future. Taken together, the mechanistic insights provided by this work may ultimately point the way to strategies for the modulation of repeat expansion and contraction that could be used for the treatment of repeat sequence associated human diseases.

## Supplementary Material

gkab573_Supplemental_File
