# Supplementary material for: Strand-specific effect of Rad26 and TFIIS in rescuing transcriptional arrest by CAG trinucleotide repeat slip-outs
Source: Nucleic Acids Res. 2021 Jul 1;49(13):7618–27. doi: 10.1093/nar/gkab573 (PMC8287942; doi:10.1093/nar/gkab573)
Supplement: gkab573_Supplemental_File [file gkab573_supplemental_file.pdf]

Strand-specific effect of Rad26 and TFIIS in rescuing transcriptional arrest by CAG trinucleotide repeat slip-outs

Jun Xu[1], Jenny Chong[1], Dong Wang[1,2,3,*]

[1]Division of Pharmaceutical Sciences, Skaggs School of Pharmacy & Pharmaceutical Sciences; University of California, San Diego, La Jolla, California 92093, United States

[2]Department of Cellular and Molecular Medicine, University of California, San Diego, La Jolla, California 92093, United States

[3]Department of Chemistry and Biochemistry, University of California, San Diego, La Jolla, California 92093, United States

SUPPORTING INFORMATION

Table of contents

Table S1. Sequences of the oligonucleotides.

1. **Figure S1.** Pol II elongation on different lengths of random sequences inserted at the same position of slip-out structures.
2. **Figure S2.** Diagram of pausing sites induced by TS slip-out.
3. **Figure S3.** Ratio of RNase H cleaved bands in TS and NTS slip-out transcription.
4. **Figure S4.** Strand-specific inhibitory effect of Spt4/5-Elf1 on split-out transcription by Pol II.
5. **Figure S5**. Rad26 and TFIIS overcome the translocation inhibitory effect of Spt4/5-Elf1.
6. **Figure S6.** Rad26 and Spt4/5 share the overlapping binding site on Pol II.

Table S1. Sequences of the oligonucleotides.

Figure	Oligo sequence: (All oligos are in 5’ to 3’ direction. Repeat regions are highlighted in orange, and perfect hairpins sequences are underlined and bolded. RNA binding site on TS is shown in bolded red).
Figure 1	**NTS:**<br>Biotin-TTTCTATATTGACTGAGTTCAGCTCATATTTATCGAGAGGAACAACGAGAGAGAGAAGTGTGTGAAGTGAGAGAG<br>TCAGCGAGGAGTAGAGAAGGAGATG-**(CAG)nx**-GAGAGGAGAGAGAGAAGAGAAGAGAGAGGCATGTCGG<br>**TS:** CCGACATGCCTCTCTCTTCTCTTCTCTCTCTCCTCTC-**(CAG)ny**-CATCTCCTTCTCTACTCCTCGCTGACTCTCTCACT<br>TCACACACTTCTCTCTCTCGTTGT**TCCTCTCGAT**AAATATGAGCTGAACTCAGTCAATATAG<br>**RNA:** AUCGAGAGGA<br>TS slip-out: n=0, nx=0, ny=0; TS slip-out n=2, nx=0, ny=2; … …; TS slip-out n=40, nx=0, ny=40;<br>NTS slip-out: n=0, nx=0, ny=0; NTS slip-out n=2, nx=2, ny=0; … …; NTS slip-out n=40, nx=40, ny=0;
Figure 2,3,4	**NTS CAG slip-out**<br>**NTS:**<br>Biotin-<br>TTTCTATATTGACTGAGTTCAGCTCATATTTATCGAGAGGAACAACGAGAGAGAGAAGTGTGTGAAGTGAGAGAGTC<br>AGCGAGGAGTAGAGAAGGAGATGCAGCAGCAGCAGCAGCAGCAGCAGCAGCAGCAGCAGCAGCAGCAGCAGCAGCAGC<br>AGCAGCAGCAGCAGCAGCAGCAGCAGCAGCAGCAGCAGCAGCAGCAGCAGCAGCAGCAGCAGCAGCAGGAGAGGAGAGAGA<br>GAAGAGAAGAGAGAGGCATGTCGG<br>**TS:**<br>CCGACATGCCTCTCTCTTCTCTTCTCTCTCTCCTCTCCATCTCCTTCTCTACTCCTCGCTGACTCTCTCACTTCACACACTT<br>CTCTCTCTCGTTGT**TCCTCTCGAT**AAATATGAGCTGAACTCAGTCAATATAG<br>**RNA:** AUCGAGAGGA<br><br>**TS CAG slip-out**<br>**NTS:**<br>Biotin-<br>TTTCTATATTGACTGAGTTCAGCTCATATTTATCGAGAGGAACAACGAGAGAGAGAAGTGTGTGAAGTGAGAGAGTC<br>AGCGAGGAGTAGAGAAGGAGATGGAGAGGAGAGAGAGAAGAGAAGAGAGAGGCATGTCGG<br>**TS:**<br>CCGACATGCCTCTCTCTTCTCTTCTCTCTCTCCTCTCCAGCAGCAGCAGCAGCAGCAGCAGCAGCAGCAGCAGCAGCAGCA<br>G<br>CAGCAGCAGCAGCAGCAGCAGCAGCAGCAGCAGCAGCAGCAGCAGCAGCAGCAGCAGCAGCAGCAGCAGCAGCAGCAT<br>CTCCTTCTCTACTCCTCGCTGACTCTCTCACTTCACACACTTCTCTCTCTCGTTGT**TCCTCTCGAT**AAATATGAGCTGAACT<br>CAGTCAATATAG<br>**RNA:** AUCGAGAGGA

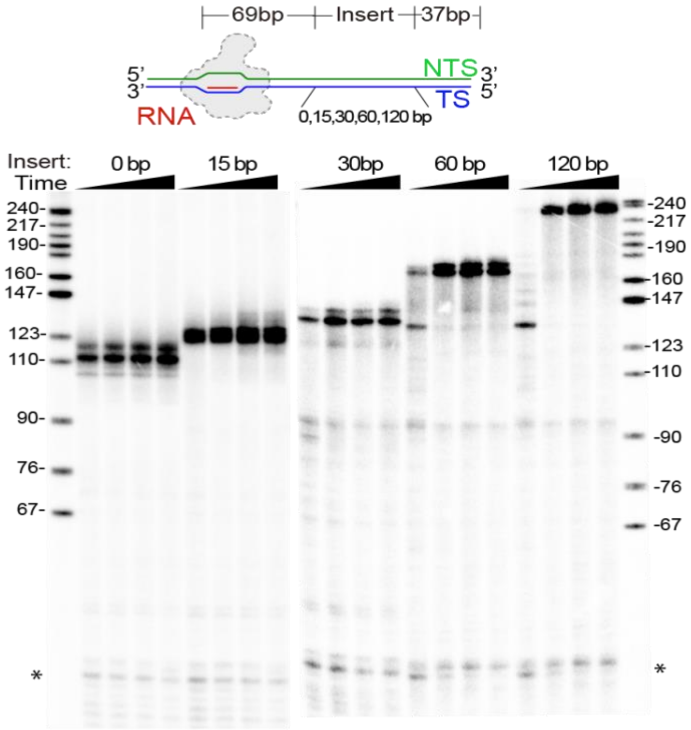

Figure S1. Pol II elongation on different lengths of random duplex DNA sequences inserted at the same position of slip-out structures. Especially, there is no obvious pausing at the slip-out induced translocation barrier region (from the ligation truncation transcript to the 76-nt maker), supporting that Pol II pausing at the region when slip-out structures were present is specifically induced by the slip-out DNA structures. Starred bands (*) indicate "ligation truncation" that corresponds to short run-off transcript from the reconstituted ECs that did not ligate to the downstream DNA. Time points are 0.3, 1, 3, and 10 min.

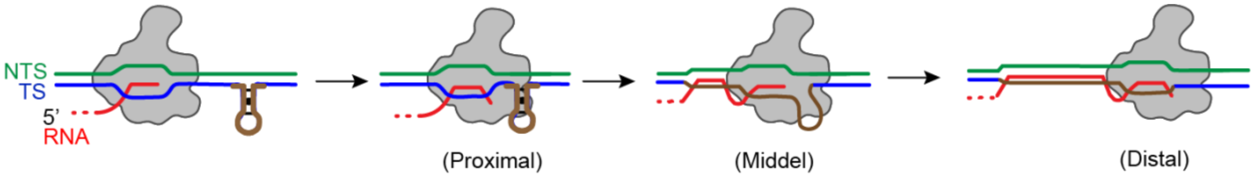

Figure S2. Diagram of pausing sites induced by TS slip-out. The TS slip-out induced Pol II pausing can be roughly divided into three regions: The proximal site, where Pol II start to contact with the slip-out structure; the distal sites, where Pol II transcribed all of the slip-out region and start to run into the matched duplex region; the middle region which is between the proximal and distal regions. The repeat region is shown in brown.

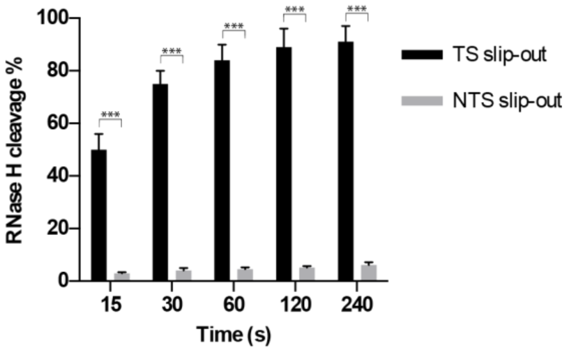

Figure S3. Ratio of RNase H cleaved bands in TS and NTS slip-out transcription. The RNase H cleavage ratio in TS slip-out is significantly higher than that of NTS. All data in this figure were obtained and quantified from three independent experiments (means ± SEM, n= 3, two-tailed Student's *t*-test, ***P < 0.001).

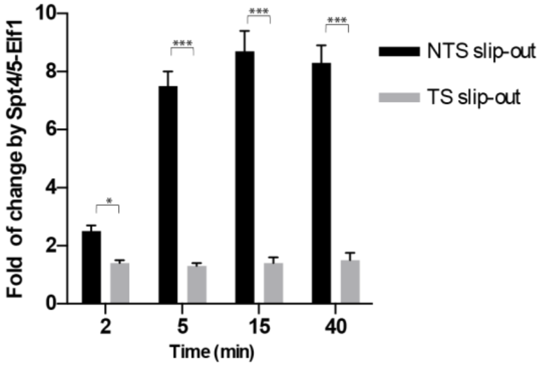

Figure S4. Strand-specific inhibitory effect of Spt4/5-Elf1 on split-out transcription by Pol II. Spt4/5-Elf1 complex has a much stronger inhibitory effect on Pol II bypass of the NTS slip-out than that of the TS slip-out. All data in this figure were obtained and quantified from three independent experiments (means ± SEM, n= 3, two-tailed Student's *t*-test, *P < 0.05, ***P < 0.001). Fold of change by Spt4/5-Elf1=run off %(buffer)/ run off %(Spt4/5-Elf1). The data is from Figure. 4.

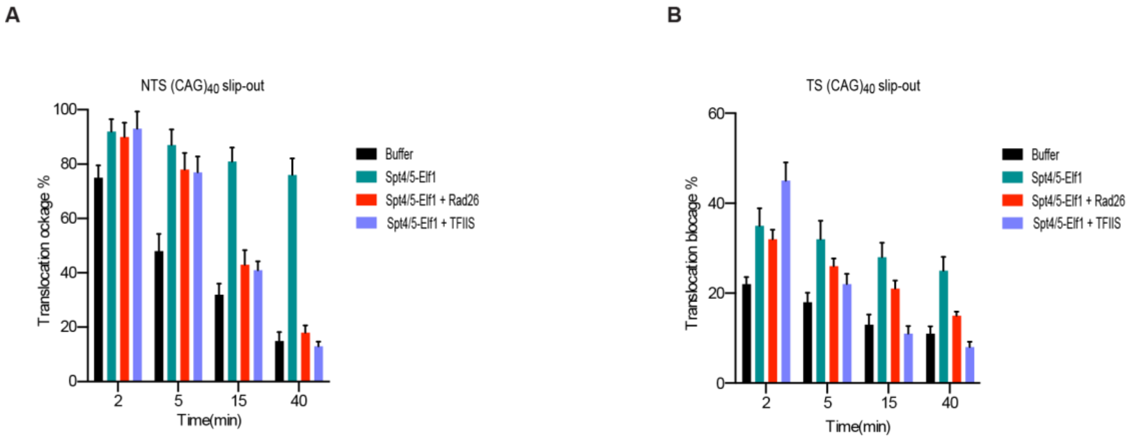

Figure S5. Rad26 and TFIIS overcome the translocation inhibitory effect of Spt4/5-Elf1. (*A*) Rad26 and TFIIS overcome the translocation inhibitory effect of Spt4/5-Elf1 and promote Pol II bypass of the NTS $(CAG)_{40}$ slip-out. (*B*) Rad26 and TFIIS overcome the translocation inhibitory effect of Spt4/5-Elf1 and promote Pol II bypass of the TS $(CAG)_{40}$ slip-out induced translocation blockage.

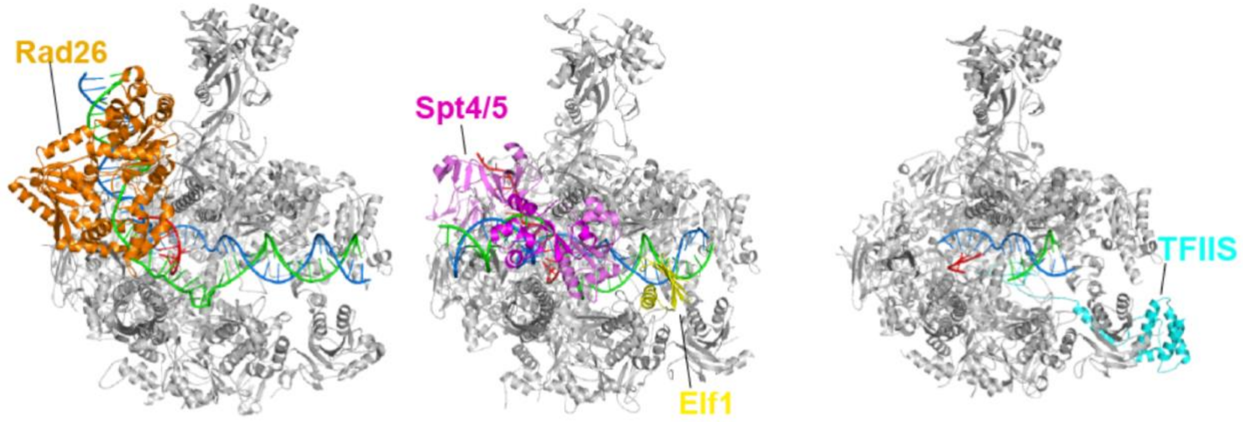

Figure S6. Rad26 and Spt4/5 share the binding site on Pol II. Structures of Pol II elongation complex (Pol II EC) with transcription elongation factors Rad26 (PDB ID: 5VVR), Spt4/5-Elf1 (PDB ID: 6J4Y; for simplicity, the downstream nucleosome was omitted). For comparison, the TFIIS-Pol II complex structure (PDB ID: 3PO3) is also included on the right.
